# A temperature reading of COVID-19 pandemic employee agility and resilience in South Africa

**DOI:** 10.4102/sajip.v47i0.1853

**Published:** 2021-07-27

**Authors:** Cristy Leask, Shaun Ruggunan

**Affiliations:** 1 Graduate School of Business, College of Law and Management, University of KwaZulu-Natal, Durban, South Africa; 2 School of Management, IT and Governance, College of Law and Management, University of KwaZulu-Natal, Durban, South Africa

**Keywords:** employee agility and resilience, COVID-19, South Africa, impact of COVID-19 on employees, gender, COVID-19 impact on organisations

## Abstract

**Orientation:**

Employee agility and resilience are central to the flourishing of employee and organisational life. The coronavirus disease 2019 (COVID-19) pandemic amplified stressors and added new challenges for employees in South Africa. The study reported here provides a temperature reading of the agility and resilience of South African employees in the context of the pandemic.

**Research purpose:**

The aim of this study was to engage in a temperature reading of South African employees’ agility and resilience during the COVID-19 pandemic.

**Motivation for the study:**

The study was motivated by the need to understand how South African employees fare in terms of their agility and resilience levels in the context of profound social and economic disruptive events such as the COVID-19 pandemic.

**Research approach/design and method:**

A cross-sectional survey design was used employing quantitative methodologies. A total of 185 permanently employed respondents from South Africa were conveniently sampled. Descriptive and inferential statistics were used to analyse the data.

**Main findings:**

Whilst respondents reported high resilience and agility capacity, the findings also suggest that respondents’ gender, age, upskilling intentions, size of employer, organisational communication and individual renewal strategies influence their resilience and agility behaviours.

**Practical/managerial implications:**

The study prompts a discussion on how practitioners can better serve the wellness agenda of organisational life during sustained periods of organisational stress.

**Contribution/value-add:**

This study extends the theoretical and practical debate on employee agility and resilience in South African context.

## Introduction

The coronavirus disease 2019 (COVID-19) pandemic has had a profound impact on the economy, with significant psychological and social effect on global populations. The scope of COVID-19 warrants a classification as a ‘grand challenge’ (Bacq, Geoghegan, Josefy, Stevenson, & Williams, 2020). Given the global state of mental health because of COVID-19, a united cooperation in research for shared data access, expertise and capacity-building is required as a response to this ‘grand challenge’.

Debates on whether to lock down cities or countries have revolved around economic concerns primarily, with less focus on the psychological and sociological consequences of these lockdowns. Different patterns of responses, including employers, employees and states, are contextually dependent on the unique cultural, economic, social and political features of these places. Hence, we find that the Asian response differs significantly from that of the United States, for example. Even within a continent, diversity of experiences and responses occurs as is the case in Europe (Balmford, Annan, Hargreaves, Altoè, & Batemen, 2020). Context, therefore, matters and our research is embedded in a South African context, which is characterised by high levels of unemployment, poverty and inequality pre-COVID-19. Some of the South African business responses to COVID-19 have been to focus on economic survival by ceasing production, re-structuring and cutting expenses, especially staff wages, which has led to significant job losses.

## Literature review

South Africa presents an interesting case study to measure employee agility and resilience levels for the following reasons. The country has a high unemployment rate when benchmarked globally. The pandemic has exacerbated this rate to almost 50% (Statistics South Africa, 2020b), making it one of the highest in the world. Each employed South African in the formal sector on an average supports up to three other adults, making it one of the highest dependency rates in the world. Compounding this are some of the highest rates of (racialised) income inequality in the world (Statistics South Africa, 2020a). South Africans experience high rates of violent crime, chronic stress, violence against women, racism and trauma, which perhaps could only be rivalled by some South American countries (Lamb, 2019; Peltzer, 2000). Prior to COVID-19, the focus of the South African government was developing entrepreneurship and the informal and small, medium and micro enterprises (SMMEs) sector as means of job creation, skills development and the economy (Urban & Ndou, 2019). COVID-19 has devastated up to 60% of this sector (Kalidas, Magwentshu, & Rajagopaul, 2020).

Whilst the COVID-19 pandemic is significant, the South African experience of the human immunodeficiency virus (HIV) and tuberculosis (TB) epidemics (which have had higher death rates), which arguably caused deeper restructuring of society, is an experience that sets it apart from many other countries. In other words, South Africans are used to economic and social hardships. The sudden and deep disruptions of the lockdown jarred an already fraught and fragile South African society.

It is against this background that we do a ‘temperature reading’ of the agility and resilience levels of permanently employed South Africans during the current pandemic. Our research design is analogous to a physical temperature reading that is conducted to determine the state of one’s physical health at a particular moment in time. We want to ascertain the levels of employee agility and resilience of employees during profound social disruption of the COVID-19 pandemic in a rapid way that gives an overall sense of the ‘well-being’ (as measured by agility and resilience levels) of South African employees. There is less focus on developing complex statistical models to determine and assess causality, and the focus is more on ascertaining an overall and quick sense of agility and resilience levels at a particular moment in time. An ‘abnormal’ reading is an indicator that something may be amiss, or even relatively normal indicators during abnormal times may allow us to conjecture as to why this may be the case. Most of the temperature reading studies were conducted outside South Africa, and inferences were made from these global studies (Alves et al., 2020; Helgeson et al., 2020).

At the time this survey, no other study assessing these measures during the pandemic was conducted. Recently, a number of COVID-19-related employee surveys have been conducted in South Africa but none has assessed agility and resilience levels of employees (Cant, 2020; Matli, 2020). Temperature reading surveys are important during times of rapid change because they point to potential areas of concern for scholars and practitioners, whilst at the same time inviting reflection on how to better refine survey and research design for future studies (Cozby & Bates, 2015). The urgency of the context also means that as scholar-practitioners, it is important for us to act quickly so that we can best position our professions to serve both organisations and employees in South Africa.

In response to the COVID-19 pandemic, employees in the formal sector have had to work from home, change work-related processes, juggle family demands, especially as they navigate the balance of multiple roles across work with personal lives. It is not surprising that a plethora of both popular and scholarly works on resilience and agility have emerged during the lockdown, as scholars and practitioners attempt to make sense of organisational and employee experiences of the pandemic (Childress, 2020; Chong, Handscomb, Williams, Hall, & Rooney, 2020; Havnen et al., 2020). COVID-19 and the resulting social distancing policy had an unprecedented negative impact on organisational financial performance globally and nationally (Ozili & Arun, 2020).

Many organisations are facing challenges of work re-structuring, re-organisation and re-pivoting so that they become fit for the purpose. Organisational survival and effectiveness depend on the ability of organisations and employees to withstand and adapt to significant challenges, that is, on their resilience. Resilience in organisations has been acknowledged as a vital competitive advantage, and as a result, research on how to promote and improve resilience amongst employees is increasing (Bardoel, Pettit, De Cieri, & McMillan, 2014; Lengnick-Hall, Beck, & Lengnick-Hall, 2011). The increased interest in employee agility and resilience stems from a wish to strengthen organisational systems and infrastructure, and to ensure organisational sustainability, which depend on the extent to which employees are able to maintain performance levels (Lengnick-Hall et al., 2011). Agility and resilience skills help employees deal with change more effectively and positively. Organisations with high levels of agility have workforces that are innovative, fast to adapt to change and flexible (Muduli, 2013).

Against this backdrop of the impact of COVID-19 on organisations and employees, time is of the essence for industrial-organisational (I-O) psychologists to urgently intervene with evidence-based solutions and thus dissuade organisations from the lure of the ‘magic bullet’ (Rotolo at al., 2018). The purpose of the current research study was to conduct a point in time temperature reading of employees’ agility and resilience to enable nuanced and tailored interventions. During this crisis, it is an opportunity for the South African I-O psychology community to contribute to understanding the ways in which the pandemic is shaping organisational life for South African employees with relevant credible, transparent and ethical research (Van Zyl & Junker, 2019).

Employee agility and resilience have gained attention in the literature through investigating organisational change and information technology (IT) system implementation (Breu, Hemingway, Strathern, & Bridger, 2002). Qin and Nembhard (2010) defined workforce agility as the ability of employees to respond strategically to uncertainty with an emphasis on its greater salience in enterprises which rely heavily on the workforce to transfer cutting-edge technologies into products. Muduli (2013) conceptualised an agile workforce as well-trained and flexible, adapting quickly and easily to new opportunities and market circumstances. Employee agility is beneficial to the organisation’s performance (Muduli, 2013) and the ability to navigate change (Warner & April, 2012). Organisational agility is considered as a contributor to a company’s success, and thus employee agility would be valued by the organisation (Podsakoff, Whiting, Podsakoff, & Blume, 2009). Thus, employee agility and resilience will be a key ingredient to enable organisations to re-organise for survival. The current context of deep disruption may be the COVID-19 pandemic, but further deep societal disruptive events will continue. These may range from further pandemics to unknown and unanticipated future societal upheavals. Measuring levels of agility and resilience during the current societal crisis may allow us to extrapolate how organisations and employees behave during times of sustained crisis. Agility and resilience are measures of how we are able to adapt to change rapidly and ‘bounce back’ from hardship experienced during disruptive and rapidly changing contexts. They are, therefore, apt measures to do a temperature reading of agility and resilience.

Previous studies on agility across business fields have suggested that employee agility is a crucial component of organisational agility. Chonko and Jones (2009) suggested that organisational agility results from the people who comprise it working together in ways that benefit the individual, the organisation and their customers. As organisations begin to transition from the initial panic stage of shutdown to adaptions to work from home, with COVID-19, this creates a new reality. There is a need to understand the impact and responses of COVID-19 and the agility and resilience capacity of permanent employees in South Africa.

During the COVID-19 pandemic, much has been written about the importance of employee resilience and agility in terms of navigating unpredicted change, absorbing and adapting the challenges (Childress, 2020; Chong et al., 2020), ways to develop resilience and agility (Neuroleadership Institute, 2020) and leadership competence development after COVID-19 (Dirani et al., 2020). Yet there is an absence of research data providing a temperature reading on the current reality of employee agility and resilience in South Africa.

## Aim of the study

The purpose of this study was to conduct a temperature reading of South African permanent employees’ agility and resilience levels. Hence, the research question guiding the study is as follows: ‘what are the employee agility and resilience levels of employees in South Africa?’

## Contribution to the field

Much has been written about the concern of mental health of global citizens and the need for urgent wellness support (Pfefferbaum & North, 2020; Xiang et al., 2020). For I-O psychology scholar-practitioners, there has been an urgent appeal to focus research within the COVID-19 pandemic on wellness, health and safety of the employees (Rudolph et al., 2020). In response to the quest, this research offers quantitative data on the current state, a ‘temperature reading’ of COVID-19 on employee agility and resilience in South Africa. Human resources and industrial organisational psychologists are uniquely positioned to provide guidance about how COVID-19 will likely impact employees and organisation by providing evidence-based recommendations for navigating the challenges and opportunities of this crisis. Whilst a temperature reading is more suggestive than conclusive in its findings, it is nonetheless an important intervention to understand a rapidly and currently evolving situation. It flags potential areas for HR and I-O scholars to hone in on to further unpack employees and agility of employees during periods of profound social disruption.

Thus, the starting point was to take a ‘temperature reading’ to determine the impact of COVID-19 on employee agility and resilience. For the purposes of this research, the constructs for agility and resilience at an individual level were based on the development, validation and practical application of an employee agility and resilience measure (Braun, Hayes, DeMuth, & Taran, 2017), as the measure was used to take the ‘temperature reading’. According to Braun et al. (2017), agility was defined as a skill of proactive rethinking or redefining to overcome obstacles. As defined by Bridges (1980) cited in Braun et al. (2017), resilience is ‘the emotional and psychological transition to change’. We suggest that before organisations embark on their post-COVID-19 recovery plan to navigate the effects of the COVID-19 pandemic, it is important to know how this pandemic affects employees in South Africa.

## Research design and data analysis

The quantitative, non-experimental research method was deemed most suitable to answer the research questions (Creswell & Creswell, 2017). Both descriptive and inferential statistics were used in the data analysis. Using the indices from the survey, the study derived mean composite scores for each of the dimensions of agility and resilience. For example, in the case of agility, the responses to the five questions measuring agility were transformed into a single mean score representing the entire dimension of agility. This technique was conducted for all seven dimensions, which allowed for further analysis. Ordinary least squares (OLS) regression analysis was conducted on the dimensions of agility and resilience (DV) and demographics, employment and organisational characteristics, impact on employees and employees’ responses to COVID-19 (IV) (Craven & Islam, 2011).

## Research participants

The population included all people permanently employed in South Africa. The sampling strategy utilised was nonprobability with a purposive design (Etikan, Musa, & Alkassim, 2016). A total of 229 respondents completed the questionnaire; once the data were cleaned, the final sample size was 185 (*N* = 185).

## Measuring instruments

The survey questionnaire comprised four parts. The researchers developed the first three parts; part 4 is a standardised measure. The four parts were as follows: (1) demographics, six questions covering age, gender, employment, education, work sector and organisational size; (2) impact of COVID-19 on employees, four questions on the impact of COVID-19 on workload, salary and feasibility to work from home and workspace; (3) employee response to COVID-19, three questions with a 1–5 Likert scale on the risk of job loss, need to outperform peers, organisational communication and one question with a nominal rating scale on upskilling because of COVID-19; and (4) the standardised instrument of Employee Agility and Resilience (Braun et al., 2017) measures 46 items with a 1–5 Likert scale across seven dimensions: agility (14 items), resilience (nine items), individual renewal (three items), collaboration (eight items), creating positive relationships (four items), openness to experience (three items) and social support (five items) (Braun at al., 2017). A measure of Cronbach’s alpha was used to describe the internal consistency and reliability of the questions used to measure the above-mentioned psychometric constructs. Permission was granted to use the Employee Agility and Resilience survey instrument (T. Braun, pers. comm., 18 May 2020).

## Research procedures

Once the LinkedIn gatekeeper letter was obtained, an application was submitted for ethical clearance from the University Research Office. The researchers placed a notice on LinkedIn, which contained information about the title, purpose, study procedures, time commitment to complete the questionnaire, the inclusion criteria used to determine eligibility, contact details for the researchers and a link to the SurveyMonkey questionnaire. Data collection was done from 25 June 2020 to 20 July 2020, and during this time period, South Africa was in lockdown level 3. All principles of anonymity, confidentiality and non-maleficence were adhered to in the study. Participation was voluntary. Respondents had to give informed consent for participating in the study and they were informed that they could withdraw from the study at any point without any consequences for themselves.

Once the survey was closed, the data were exported and analysed using Stata software program.

### Ethical considerations

Ethical approval was obtained from the University of KwaZulu-Natal Humanities and Social Sciences Research and Ethics committee (ethical clearance number: HSSREC/00001423/2020).

## Results

### Demographics

The majority of the sample comprised women (55.68%), within the age group of 36–45 years (39.46%), living in KwaZulu-Natal (62.19%) and had some form of tertiary education (49.46%). The demographic, employment and organisational characteristics of the sample are presented in Table 1.

**TABLE 1:** Demographic, employee and organisational characteristics of the sample.

Characteristics	*f*	%
**Demographic characteristics**		
**Gender**		
Male	82	44.32
Female	103	55.68
**Total**			**185**	**100.00**
**Age (years)**		
18–25	10	5.41
26–35	57	30.81
36–45	73	39.46
46–55	33	17.84
56–65	11	5.95
66+	1	0.54
**Total**			**185**	**100.00**
**Province**		
Eastern Cape	3	1.62
Free state	1	0.54
Gauteng	42	22.70
KwaZulu-Natal	115	62.16
Mpumalanga	2	1.08
Limpopo	2	1.08
Northern Cape	0	0.00
North West	1	0.54
Western Cape	19	10.27
**Total**			**185**	**100.00**
**Educational level**		
Did not complete school	3	1.63
Completed Grade 12	29	15.76
Tertiary education	91	49.46
Master’s degree or PhD	61	33.15
No schooling	0	0.00
**Total**			**184**	**100.00**
**Employee and organisational characteristics**		
**Employment position**		
Admin	19	10.27
Staff member	77	41.62
Manager	67	36.22
Director	17	9.19
Owner	4	2.16
Other	1	0.54
**Total**			**185**	**100.00**
**Employment sector**		
Security/Police	5	2.70
Marketing	15	8.11
Sales	10	5.41
State-owned enterprise	2	1.08
Government	6	3.24
Mining	5	2.70
Agriculture, fishing and forestry	2	1.08
Financial services	25	13.51
Medical and healthcare	15	8.11
Information technology	10	5.41
Construction	7	3.78
Professional services	27	14.59
Manufacturing	14	7.57
Education	42	22.73
**Total**			**185**	**100.00**
**Organisational size**		
1–50	52	28.11
51–200	24	12.97
201–500	20	10.81
501–1000	14	7.57
1001–5000	32	17.30
5001+	41	23.24
**Total**			**185**	**100.00**
**Essential services**		
Yes	107	57.84
No	78	42.16
**Total**			**185**	**100.00**

### Impact of COVID-19 on employees

As part of a temperature research design, we were interested in whether respondents experienced any changes to their salaries and workloads. Additionally, with mandatory work from home policies being the norm during the pandemic, we wanted to assess constraints to remote working (if any). As such questions we designed were questions probing these areas. This section reports on the responses of those surveys on the impact of the pandemic on their workload, salaries and working from home constraints.

Most respondents (69.2%) had experienced an increased, moderate and low increase in workload. Of the sample, 61.08% reported that the pandemic had no impact on their salaries. Of those individuals who reported that there was an impact on their salary (38.92%), 72.22% experienced a general pay reduction. The majority of respondents (76.67%) reported that it was feasible for them to work from home. Of those that reported constraints to working from home, the majority (50.88%) of the sample indicated that resources were a constraint, whilst 15.79% experienced space constraints and 17.54% experienced constraints related to privacy. Table 2 shows the results of the impact of COVID-19 on employees.

**TABLE 2:** The impact of COVID-19 on employees.

Variables	*f*	%
**Impact of COVID-19 on workload**		
Increased	84	45.41
Moderate increase	34	18.38
Low impact increase	10	5.41
Decrease	37	20.00
No impact	20	10.81
**Total**	**185**	**100.00**
**Impact of COVID-19 on salary**		
Yes	72	38.92
No	113	61.08
**Total**	**185**	**100.00**
**Type of impact on salary**		
General pay reduction	39	72.22
Reduced work hours	3	5.56
Reduction in output (manufacturing)	1	1.85
Increase in pay	1	1.85
Bonus, benefits and leave reduction	6	11.11
Complete loss of income	4	7.41
**Total**	**54**	**100.00**
**Feasible to work from home**		
Yes	142	76.76
No	43	23.24
**Total**	**185**	**100.00**
**Constraints to work from home**		
Space	9	15.79
Resources	29	50.88
Privacy	10	17.54
Other	9	15.79
**Total**	**57**	**100.00**
**Other constraints**		
I have to work onsite	6	75
Prohibited because of lockdown regulations	2	25
**Total**	**8**	**100.00**

COVID-19, coronavirus disease 2019.

### Employee responses to COVID-19

In this section, we report on employee responses to questions on perceptions of their job security, the pressure to outperform their peers to demonstrate their usefulness to their employers, the extent to which they were informed of organisational changes and whether they had considered upskilling as a strategy to demonstrate value to their organisations.

The results show that 44.51% (strongly disagree and disagree) of respondents feel that they are not at risk of losing their job, whilst 34.06% of respondents agreed that they need to outperform their peers to survive in their respective jobs or organisations. An overwhelming majority of respondents (67%) stated that they are well informed of organisational changes. More than 42% of respondents have considered upskilling in response to COVID-19, whilst 29.12% of the sample indicated that they have already started upskilling. The results are shown in Table 3.

**TABLE 3:** Employee responses to COVID-19.

Variables	*f*	%
**Risk of losing job because of COVID-19**		
Strongly disagree	44	24.18
Disagree	37	20.33
Neutral	52	28.57
Agree	30	16.48
Strongly agree	19	10.44
**Total**	**182**	**100.00**
**Outperform peers for survival**		
Strongly disagree	40	21.98
Disagree	36	19.78
Neutral	44	24.18
Agree	32	17.58
Strongly agree	30	16.48
**Total**	**182**	**100.00**
**Well informed of changes**		
Strongly disagree	17	9.34
Disagree	8	4.40
Neutral	35	19.23
Agree	49	26.92
Strongly agree	73	40.11
**Total**	**182**	**100.00**
**Upskilling in response to COVID-19**		
Yes	78	42.86
No	51	28.02
I have already started upskilling	53	29.12
**Total**	**182**	**100.00**

COVID-19, coronavirus disease 2019.

### Employee agility and resilience

The purpose of this section is to report how respondents scored on the dimensions of agility and resilience. Furthermore, we report the correlations between each of the dimensions of agility and resilience. The section ends with a reportage of the mean scores of agility and resilience.

The correlations of each of the dimensions of agility and resilience are shown in Table 4. The results indicate that there is a medium correlation between agility and collaboration (*r* = 0.51, *p* < 0.05), a medium correlation between agility and resilience (*r* = 0.48, *p* < 0.05) and resilience and collaboration (*r* = 0.55, *p* < 0.05), with a very weak correlation between agility and individual renewal (*r* = 0.02). The results show that the mean agility score for the sample is fairly agile (*M* = 20.11) with the lowest dimension of individual renewal (*M* = 10.22), as shown in Table 5. The results suggest that the sample, on an average, score is higher on all the dimensions of agility and resilience.

**TABLE 4:** Correlation between the dimensions of agility and resilience.

Variables	Mean	Standard deviation	1	2	3	4	5	6	7
Agility	20.11	2.97	0.78	-	-	-	-	-	-
Resilience	24.46	3.24	0.48*	0.83	-	-	-	-	-
Collaboration	20.57	2.58	0.51*	0.55*	0.74	-	-	-	-
Creating positive relationships	25.84	3.03	0.36*	0.45*	0.52*	0.86	-	-	-
Social support	19.80	3.35	0.20*	0.25*	0.37*	0.50*	0.81	-	-
Individual renewal	10.22	2.80	0.02	0.14	0.13	0.15	0.24*	0.77	-
Openness to experience	12.42	1.50	0.30*	0.42*	0.42*	0.43*	0.23*	0.15*	0.70

Note: Figures in parenthesis are internal reliability estimates (Cronbach’s alpha).

* , Denotes a statistical significance at 95% level of confidence (*p* < 0.05).

**TABLE 5:** Scores of agility and resilience.

Variables	Mean score	Standard error	Min	Max	*f*
Agility	20.11	0.227	5	25	171
Resilience	24.46	0.248	6	30	171
Collaboration	20.57	0.197	5	25	171
Creating positive relationships	25.84	0.232	6	30	171
Social support	19.80	0.256	5	25	171
Individual renewal	10.22	0.215	3	15	169
Openness to experience	12.42	0.115	3	15	171

#### Regression model

A regression model was used to determine the relationship between the dimensions of employee agility and resilience scores and each of the demographic variables, as shown in Table 6. The results showed that gender is not a significant predictor of agility. Employees aged 56–65 years are significantly less agile than those aged 36–45 years (−2.2069, *p* < 0.05). Directors are significantly more resilient than staff members (2.6487, *p* < 0.05). Regarding organisational size, employees who work in organisations with between 51 and 200 staff members are significantly more resilient than those who work in organisations with 0–50 staff members (1.9823, *p* < 0.05). Women are significantly less likely to participate in individual renewal activities than their male counterparts (−1.2851, *p* < 0.05). The results show that employment, organisational size and impact of COVID-19 on employees are significant predictors of the various dimensions of agility and resilience.

**TABLE 6:** Regression of predicting the relationship between dimensions of employee agility and resilience and demographics and impact of COVID-19 on employees.

Variables	Agility		Resilience		Collaboration		Creating positive relationships		Social support		Individual renewal		Openness to experience	
	Mean	Standard error	Mean	Standard error	Mean	Standard error	Mean	Standard error	Mean	Standard error	Mean	Standard error	Mean	Standard error
**Gender**														
Female	−0.1593	0.4398	0.4398	0.5524	0.0308	0.4484	−0.2110	0.5026	−0.2897	0.5927	−1.2851**	0.4958	−0.0449	0.2834
**Age categories**														
18–25 years	−0.6615	1.0968	2.1343	1.3777	0.7559	1.1182	1.0176	1.2534	0.1743	1.4781	−0.4429	1.2181	0.3144	0.7067
26–35 years	−0.3663	0.5466	0.3419	0.6866	−0.1664	0.5573	1.8046***	0.6247	0.8242	0.7367	0.4386	0.6130	0.3236	0.3522
46–55 years	−0.2694	0.6071	0.4902	0.7626	0.1154	0.6190	0.7669	0.6938	0.6923	0.8182	0.4630	0.6799	0.1309	0.3912
56–65 years	−2.2069**	1.0596	1.1983	1.3310	0.2043	1.0803	2.1526*	1.2109	0.3084	1.4280	1.1284	1.1771	0.1440	0.6828
**Province**														
Eastern Cape	2.4345	1.6834	2.5753	2.1146	2.0298	1.7162	2.4782	1.9238	−3.5759	2.2687	−3.1750*	1.8704	0.9379	1.0847
KwaZulu-Natal	1.2695**	0.6098	0.5341	0.7660	0.4737	0.6217	1.4047**	0.6969	0.4653	0.8218	−0.0580	0.6783	0.4942	0.3929
Mpumalanga	−0.7742	2.1891	−1.3745	2.7498	−0.3064	2.2318	1.0718	2.5017	−4.2471	2.9502	−0.9630	2.4342	0.0982	1.4106
Limpopo	0.9767	3.0386	1.5101	3.8170	2.7742	3.0979	−1.3444	3.4726	2.5055	4.0951	−0.0684	3.3794	0.6528	1.9580
North West	0.5821	2.9184	−3.7233	3.6659	−3.8567	2.9753	0.2567	3.3352	−0.9865	3.9330	−4.4574	3.2442	−0.4762	1.8805
Western Cape	1.4156	0.8979	−0.5551	1.1279	0.2560	0.9154	1.1363	1.0261	−0.8290	1.2100	−0.1220	1.0008	0.4546	0.5786
**Educational level**														
Did not complete school	−1.2368	2.7653	4.8127	3.4736	−2.0303	2.8192	1.0006	3.1602	0.0786	3.7267	0.3714	3.0812	0.4714	1.7819
Completed Grade 12	0.6242	0.6470	0.4988	0.8128	1.1312*	0.6597	0.5636	0.7394	1.1484	0.8720	−0.9301	0.7199	−0.0978	0.4169
Master’s degree or PhD	0.7538	0.5138	0.0645	0.6454	−0.0588	0.5238	0.6503	0.5872	−0.5924	0.6925	0.2225	0.5752	0.4772	0.3311
**Work position**														
Admin	0.2011	0.8228	−0.1111	1.0335	−0.8236	0.8388	1.7374*	0.9403	−1.4018	1.1089	−0.0988	0.9149	0.1573	0.5302
Manager	1.6647***	0.5380	0.5294	0.6758	0.2252	0.5485	1.0234*	0.6149	0.5068	0.7251	−0.4999	0.5994	0.2304	0.3467
Director	4.7180***	0.9147	2.6487**	1.1490	1.8505**	0.9325	4.2742***	1.0453	0.0792	1.2327	−1.3858	1.0476	0.7668	0.5894
Owner	−0.2658	1.5198	3.0972	1.9091	0.6650	1.5495	2.2109	1.7369	−2.7419	2.0482	−2.3040	1.6885	0.6114	0.9793
**Employment sector**														
Security/Police	3.9175***	1.4773	2.7733	1.8557	2.6781*	1.5061	3.9549**	1.6883	2.3026	1.9909	−0.9080	1.6423	1.0091	0.9519
Marketing	−0.4562	0.8939	−2.1899*	1.1229	−1.8350**	0.9114	1.6370	1.0216	−1.3463	1.2047	−1.1438	0.9957	0.3712	0.5760
Sales	−0.4883	1.1120	1.5490	1.3968	0.0608	1.1337	−2.3828*	1.2708	−3.9116**	1.4986	0.9433	1.2365	0.7917	0.7165
State-owned enterprise	3.2717*	1.9515	−0.2891	2.4514	−0.8608	1.9896	0.3777	2.2302	−5.9440**	2.6300	2.3685	2.1705	−0.2028	1.2575
Government	2.2984	1.5166	−0.9064	1.9050	−1.1920	1.5462	0.2810	1.7332	0.0643	2.0439	0.7910	1.6881	0.2677	0.9772
Mining	2.0637	1.5241	2.0348	1.9145	1.7699	1.5538	1.5379	1.7418	−0.3834	2.0540	1.1778	1.6948	1.2265	0.9821
Agriculture, Fishing and Forestry	−6.5540**	2.8099	−0.8477	3.5296	−3.9136	2.8647	−6.1797*	3.2112	−3.1227	3.7868	1.1504	3.1198	−0.6279	1.8106
Financial services	0.1586	0.9022	−1.0198	1.1333	−0.8302	0.9198	0.2538	1.0311	−0.1474		1.2159	1.0091	0.4699	0.5813
Medical and healthcare	0.1299	0.9349	0.1325	1.1744	−0.5538	0.9531	−0.6451	1.0684	−2.5317**		1.2599	1.0456	0.1126	0.6024
Information technology	−1.1020	1.0976	−1.1014	1.3788	−0.0620	1.1190	1.6266	1.2544	−0.0731	1.4792	1.9447	1.2228	0.4305	0.7073
Construction	0.6342	1.2426	0.1232	1.5609	0.9694	1.2669	0.0100	1.4201	−1.2652	1.6747	2.3933*	1.3824	−0.1400	0.8007
Professional services	−0.2504	0.7746	−0.0326	0.9731	0.0885	0.7898	0.0341	0.8853	−0.4685	1.0440	0.6083	0.8662	0.7710	0.4992
Manufacturing	1.7241*	1.0194	1.2396	1.2806	0.6528	1.0393	0.6806	1.1650	−0.0482	1.3739	1.2999	1.1365	0.1156	0.6569
**Organisational size**														
51–200	0.6274	0.7850	1.9823**	0.9861	0.9974	(0.8003	1.8525**	0.8971	0.8574	1.0580	0.2719	0.8824	0.3888	0.5058
201–500	0.6052	0.7958	1.5433	0.9997	0.7216	0.8114	−0.1003	0.9095	0.2834	1.0725	0.6182	0.8874	0.2823	0.5128
501–1000	0.4194	0.9458	0.1388	1.1881	0.4042	0.9643	0.0206	1.0809	−0.9304	1.2747	0.7767	1.0506	−0.2384	0.6095
1001–5000	−0.2673	0.7242	0.9417	0.9097	−0.2012	0.7383	2.5682***	0.8276	0.4944	0.9760	1.0656	0.8042	0.6436	0.4667
5001+	−0.1325	0.7107	1.7906**	0.8927	0.3661	0.7245	1.6957**	0.8122	0.7156	0.9578	0.5030	0.7906	0.6899	0.4579
**COVID-19 characteristics**														
Not essential services	−0.2864	0.5339	0.9676	0.6707	0.2471	0.5443	0.2678	0.6102	0.2639	0.7195	1.0568*	0.5997	0.3039	0.3440
Moderate increase in workload	−0.1731	0.6080	−0.9232	0.7638	−0.8994	0.6199	−0.4544	0.6949	0.7841	0.8195	−0.5474	0.6890	−0.1475	0.3918
Low impact increase on workload	−0.1402	0.9698	0.1111	1.2182	0.6680	0.9887	−0.3785	1.1083	1.8626	1.3070	2.7230**	1.1455	0.5886	0.6249
Decreased workload	−1.1754*	0.6803	−1.2818	0.8545	−0.6862	0.6936	−1.2199	0.7774	0.9942	0.9168	1.0992	0.7570	0.2322	0.4384
No impact on workload	−2.2512***	0.7356	−0.2310	0.9241	−1.1326	0.7500	−0.3901	0.8407	0.4742	0.9914	0.0916	0.8185	0.1494	0.4740
No impact on salary	−1.7331***	0.5281	−0.6303	0.6634	−0.5667	0.5384	−1.5663**	0.6035	−0.2020	0.7117	1.0115*	0.5882	−0.3274	0.3403
Not feasible to work from home	1.7517*	1.0203	−0.4890	1.2816	−0.0270	1.0402	0.4588	1.1660	0.9810	1.3750	0.0253	1.1330	−0.5464	0.6574
**Constraints to work from home**														
Space	0.1970	1.4120	1.6302	1.7737	−1.0315	1.4396	−0.0109	1.6137	−2.9798	1.9030	0.9005	1.5698	−0.6607	0.9099
Resources	−1.9357*	1.1510	−1.7659	1.4459	−0.0994	1.1735	0.1721	1.3154	−0.2590	1.5512	−0.2322	1.2826	0.1276	0.7417
Privacy	1.9670	1.3232	3.9725**	1.6622	2.8393**	1.3490	3.9016**	1.5122	3.0522*	1.7833	0.4156	1.4700	2.0538**	0.8526
**Constant**	19.3791***	1.1804	22.9388***	1.4827	20.2565***	1.2034	22.2869***	1.3489	19.1460***	1.5908	9.0502***	1.3127	10.9322***	0.7606
**Observations**	169	-	169	-	169	-	169	-	169	-	167	-	169	-
***R*-squared**	0.5052	-	0.3403	-	0.3100	-	0.3814	-	0.2934	-	0.3222	-	0.2036	-

COVID-19, coronavirus disease 2019.

Omitted categories: males, age 36–45 years, Gauteng, tertiary education, staff member, education, organisational size 1–50, essential service, increased workload, feasible to work from home, no space constraint, no resource constraint and no privacy constraint.

Significance levels:

* , *p* < 0.1;

** , *p* < 0.05;

*** , *p* < 0.01.

Regression analysis was conducted to determine the relationship between dimensions of employee agility and resilience and employee responses to COVID-19, as shown in Table 7. The regression models in Table 7 test the relationships between the dimensions of agility and resilience and the various employee responses to COVID-19. The results show that individuals who believe that they are at risk of losing their jobs because of COVID-19 have significantly less social support than those who are neutral to their perception of job loss (−1.6154, *p* < 0.05), and those who feel that they are at risk of losing their jobs are also significantly less likely to spend time in individual renewal than their counterparts (−1.3906, *p* < 0.05). The results show that those who are well informed about organisational changes are significantly more resilient, create more positive relationships and are more open to experience than those who reported being neutral about being well informed (1.4163, 1.5327 and 0.6498, respectively, *p* < 0.05). Individuals who have already started upskilling have more social support and are more open to experience than their counterparts who have not thought about upskilling because of COVID-19 (1.3311 and 0.6077, respectively, *p* < 0.05).

**TABLE 7:** Regression estimation predicting the relationship between dimensions of employee agility and resilience and employee response to COVID-19.

Variables	Agility		Resilience		Collaboration		Creating positive relationship		Social support		Individual renewal		Openness to experience	
	Mean	Standard error	Mean	Standard error	Mean	Standard error	Mean	Standard error	Mean	Standard error	Mean	Standard error	Mean	Standard error
**Risk of job loss because of COVID-19**														
Disagree	0.1613	0.5865	0.6404	0.6385	0.0377	0.5037	0.2110	0.5913	−0.8114	0.5820	−1.5748***	0.5513	−0.3378	0.2927
Agree	0.8742	0.6415	0.4030	0.6984	0.9431*	0.5509	0.1967	0.6467	−1.6154**	0.6365	−1.3906**	0.5987	0.3312	0.3201
**Well informed of organisational changes**														
Disagree	0.1823	0.8246	0.4504	0.8978	0.5501	0.7082	1.0585	0.8314	3.6831***	0.8183	0.0668	0.7696	0.2425	0.4115
Agree	0.9896	0.6323	1.4163**	0.6884	1.4186***	0.5430	1.5327**	0.6375	3.5743***	0.6274	0.0943	0.5903	0.6498**	0.3156
**Outperform peers**														
Disagree	−0.1098	0.6199	0.9505	0.6749	0.6574	0.5324	0.9616	0.6249	0.3945	0.6151	−0.1352	0.5863	0.3730	0.3093
Agree	−0.0452	0.6462	1.1976*	0.7035	0.2298	0.5550	0.5151	0.6515	−0.7583	0.6412	−1.0340*	0.6074	0.2216	0.3225
**Upskilling in response to COVID-19**														
No	−0.8482	0.5819	−0.3542	0.6335	−0.3260	0.4997	−0.6255	0.5866	0.7492	0.5774	0.0853	0.5476	0.1985	0.2904
Already started upskilling	0.3065	0.5426	0.6586	0.5908	0.4140	0.4660	0.7752	0.5471	1.3311**	0.5384	0.3594	0.5101	0.6077**	0.2708
**Constant**	19.2850***	0.8748	22.0666***	0.9524	18.8296***	0.7513	23.8035***	0.8820	17.0865***	0.8680	11.5328***	0.8194	11.5246***	0.4366
**Observations**	171	-	171	-	171	-	171	-	171	-	169	-	171	-
***R*-squared**	0.0605	-	0.0652	-	0.0808	-	0.0826	-	0.2746	-	0.0792	-	0.0828	-

COVID-19, coronavirus disease 2019.

Significance levels:

* , *p* < 0.1;

** , *p* < 0.05;

*** , *p* < 0.01.

Omitted categories: risk of job loss – neutral, well informed – neutral, outperform peers – neutral and upskilling in response to COVID-19 – yes.

## Discussion

### Explaining high employee agility and resilience levels

This section offers possible explanations for the relatively high levels of resilience and agility of the study sample. We report first on the levels of resilience and agility and offer six possible reasons for why, despite the various challenges of the pandemic on workers globally, South African employees scored relatively high on both agility and resilience scores.

The findings show that out of a lowest possible score of 5 and a highest possible score of 25, the mean agility score was 20.11 and the mean resilience score was 24.46. These results were unexpected given the literature showing that employees are struggling on both these measures, especially in Europe and North America (Barzilay et al., 2020; Hite & McDonald, 2020). This presents a challenge to our conjecture that the hardships of the pandemic would impact negatively employees’ levels of agility and resilience. The first possible reason for this may be that extant pre-pandemic social, political and economic hardships faced by South Africans have an inoculating effect on their resilience and agility levels.

For example, research has shown that South Africans are more ‘can-do’ in terms of their attitude, enjoy high levels of resilience and are globally employees of choice (The South African, 2016). Another example of this can be found in a recent survey of South African millennials. Millennials in the survey scored higher on the mood index than their global counterparts (Deloitte Consulting, 2020). A second possibility in explaining the relatively high levels of resilience and agility may be related to the nature of the sample. The fact that the sample size was a cohort of permanently employed South Africans could mean that they felt relatively secure in their jobs, and in an attempt to maintain this security, they were willing and able to adapt to new work organisation demands.

Thirdly, the majority of participants were from the education (23%) and professional services sector (14%) with high levels of tertiary education. These are sectors that require to have relative stability during the pandemic in terms of job security. Employees in these sectors would also be highly credentialed, which may give them a sense of being less vulnerable in the labour market. Additionally, the nature of the education and professional services sectors also mean that they are better positioned to catalyse employee’s agility and resilience levels through training, development and support initiatives.

Fourthly, over 45% of the participants were in management positions. Cumulatively, this meant that this cohort of the sample were highly credentialed and experienced in organisational life and therefore better prepared to adapt and cope with organisational change. They would have had prior experiences of organisational and personal stress as well as methods to mitigate this. South Africa’s pre-pandemic normative context of uncertainty in the economy characterised by high unemployment, rolling power outages, adversarial labour relations framework and prior HIV and TB epidemics would also have had some influence on honing employee’s agility and resilience levels. Organisational learning during these conditions would have served a protective function despite the amplification of restructuring and remote working policies, especially. Agility traits such as the ability to assess the dynamics of the workplace and the ability to adapt to organisational change could be the outcome of prior organisational learning experiences.

Fifthly, literature suggests that respondents may score themselves higher on these dimensions if it reflects positively on their perceptions of self-worth. Finally, 58% of our sample identified themselves as essential workers. Employees in essential services are less likely to be retrenched or fired. This, therefore, may have also impacted the high agility and resilience scores, given that essential workers would feel greater job security than those not classified as essential during the pandemic.

### Employees’ responses to COVID-19

The previous section offered conjectures as to why respondents scored relatively high resilience and agility levels. This section analyses the sample’s responses with regard to gender, age, size of organisation and work organisation.

### Gender, agility and resilience

The results show that gender is not a significant predictor of agility. Importantly, the analysis showed that women are significantly less likely to participate in individual renewal activities than males. Individual renewal is the behavioural action of self-care, where breaks are taken to build and renew capacity. According to Gill and Orgad (2018), women are under increased social pressure to be agile and resilient in order to be perceived as successful. The extra gendered (child care, emotional labour, domestic work, and negotiating patriarchy on a daily basis) work that women engage in often builds their resiliency and agility levels, more so than men. This finding challenges the perception that women are more likely to engage in self-care activities than men, because self-care is stereotypically viewed as a feminine activity. However, it also implies that women are most likely to engage in care activities of others including partners and children at the expense of their own care. Studies have shown that women are disproportionately bearing the brunt of the remote work policies of organisations during the pandemic (Power, 2020). This double burden on women is regardless of their rank at work (Alon, Doepke, Olmstead-Rumsey, & Tertilt, 2020; Minello, 2020). This has implications for how and where psychological support is offered in organisations, especially as most organisations are focusing on gender equality and inclusion.

### Age, agility and resilience

Our study further shows that individuals aged 56–65 years are significantly less agile than those aged 36–45 years (−2.2069, *p* < 0.05). This is in line with studies that showed that younger people tend to be more agile and resilient (Muduli, 2013). Older employees may be less open to change and have fewer support mechanisms for resilience despite greater experiential learning.

### Organisational size, agility and resilience

Respondents who work in organisations with between 51 and 200 staff members are significantly more resilient than those who work in organisations with 0–50 staff members (1.9823, *p* < 0.05). Moreover, individuals who work in organisations with 5001 or more staff members have a significantly higher resilience than those who work in organisations with 0–50 staff members (1.7906, *p* < 0.05). This is in line with the conjecture that larger organisations offer more tangible support for all dimensions of agility and resilience, especially collaboration, support, networking and resilience training. Larger organisations are more likely to have dedicated human resources functions that facilitate employee resilience and agility, such as employee wellness programmes or dedicated training opportunities for staff. The concern, however, then remains for small to medium enterprises (SMMEs) that may not have the resources or know how to support mental and emotional well-being of employees. Given the centrality of the SMME sector for South Africa’s economic growth, this is an area of concern.

### Impact on work organisation

With regard to impact on employment, our results show that the majority of respondents (45%) experienced a high increase in workload. This is in line with similar studies conducted globally (Kramer & Kramer, 2020). At the same time, 20% of respondents reported a decrease in workload. The inclination to upskill may be related to increased workload across multiple skill sets. Surprisingly, only 39% of employees experienced an impact on their salaries, but this may be related to the nature of the sectors they work in. Impact on salary mainly consisted of a general pay reduction. The majority of the sample (77%) indicated that they were able to work from home despite the constraints of resources, privacy and space. The largest constraint reported was lack of resources.

Given the temperature reading design of the study, we aimed to obtain an empirical ‘snapshot’ of the agility and resilience levels of employees as well as to report on their responses to questions on work organisation during the pandemic. This section has focused on four key areas of gender, age, organisation size and the impact of workload which inform tailored practical implications for I/O psychologists.

## Limitations

As with all research, this study has limitations and the following should be taken into account when reviewing the research results. The convenience sample that was used to gather self-report data from permanent employees via LinkedIn was biased to professional employees within the KwaZulu-Natal province. A further limitation was the self-report measures, which may have impacted the results as the sample may have given favourable responses. Nevertheless, the study provides useful and important insights, a ‘temperature reading’, into the impact of COVID-19 on employee agility and resilience in South Africa. In addition, caution should be made to generalise these findings as the COVID-19 circumstances are different in each country.

As this was an exploratory ‘temperature reading’ study, future research should apply more representative sampling techniques to access a generalisable sample of the general population. As organisations enter into the COVID-19 recovery strategy, there will be merit in adding measures of employee wellness to track the individual renewal dimension as well as leadership and organisational culture as mediating and moderating variables, ultimately, in a longitudinal research study.

## Implications for the profession

The high-level practical implications are to use the ‘temperature reading’ results to raise awareness of employees’ own agility and resilience and then build interventions, especially in the COVID-19 employee-care and organisation recovery plan. Recently, there has been a dearth of resilience-building self-help; in the absence of organisational authentic empathy, these self-help initiatives can perpetuate the ‘us and them divide’, which exists in some organisations. Based on these results, the specific practical applications would be to:

- Continue to communicate to employees as this has a significant impact on resilience, collaboration, creating positive relationships.
- Support women employees with tailored individual renewal activities.
- Utilise the age group of 26–35 years employees to expand positive relationships across organisations.
- Set up resilience mentor collaborative partnerships, the larger organisations, especially, directors, who have high resilience scores to partner with organisations of size 1–200. This is a vital offering, especially given the large number of SMME businesses in South Africa.

## Conclusions

The overarching purpose of this article was to determine employee agility and resilience capacity during the COVID-19 pandemic and understand employees’ responses to the pandemic in South Africa. The research findings contribute to the knowledge base and practice of I-O psychology. These results add value for leaders, I-O psychologists and HR professionals who are either reviewing or starting their post-COVID strategies, highlighting the vital focus on tailored employee agility and resilience solutions with partnerships. HR and I-O professionals are in important positions within academia and organisations in South Africa in order to design and implement employee agility and resilience evidence-based initiatives using empirical data.
